# Clinical Implications of Diffuse Excessive High Signal Intensity (DEHSI) on Neonatal MRI in School Age Children Born Extremely Preterm

**DOI:** 10.1371/journal.pone.0149578

**Published:** 2016-02-17

**Authors:** Lina Broström, Jenny Bolk, Nelly Padilla, Béatrice Skiöld, Eva Eklöf, Gustaf Mårtensson, Brigitte Vollmer, Ulrika Ådén

**Affiliations:** 1 Department of Women's and Children's Health, Karolinska Institutet, Stockholm, Sweden; 2 Neonatal Unit, Sachs’ Children and Youth Hospital, Stockholm, Sweden; 3 Neonatal Unit, Karolinska University Hospital, Stockholm, Sweden; 4 Clinical Neurosciences, Clinical and Experimental Sciences, University of Southampton, Southampton, United Kingdom; The Research Institute at Nationwide Children's Hospital, UNITED STATES

## Abstract

**Objective:**

Magnetic resonance imaging (MRI) of the brain carried out during the neonatal period shows that 55–80% of extremely preterm infants display white matter diffuse excessive high signal intensity (DEHSI). Our aim was to study differences in developmental outcome at the age of 6.5 years in children born extremely preterm with and without DEHSI.

**Study Design:**

This was a prospective cohort study of 83 children who were born in Stockholm, Sweden, between 2004 and 2007, born at gestational age of < 27 weeks + 0 days and who underwent an MRI scan of their brain at term equivalent age. The outcome measures at 6.5 years included testing 66 children with the modified Touwen neurology examination, the Movement Assessment Battery for Children 2, the Wechsler Intelligence Scale for Children—Fourth Edition, Beery Visual-motor Integration test—Sixth Edition, and the Strengths and Difficulties Questionnaire. Group-wise comparisons were done between children with and without DEHSI using Student t-test, Mann Whitney U test, Chi square test and regression analysis.

**Results:**

DEHSI was detected in 39 (59%) of the 66 children who were assessed at 6.5 years. The presence of DEHSI was not associated with mild neurological dysfunction, scores on M-ABC assessment, cognition, visual-motor integration, or behavior at 6.5 years.

**Conclusion:**

The presence of qualitatively defined DEHSI on neonatal MRI did not prove to be a useful predictor of long-term impairment in children born extremely preterm.

## Introduction

Extremely preterm children (born before 28 weeks of gestation) are born during a vulnerable stage of brain development and injuries are common, especially in the white matter [1, 2]. Although the incidence of focal periventricular lesions has decreased, non-focal white matter abnormalities are seen in a high proportion of preterm infants using conventional structural magnetic resonance imaging (MRI) scans [1]. Diffuse excessive high signal intensity (DEHSI) is defined as increased signal intensity in the periventricular and subcortical white matter on T2-weighted MRI around term equivalent age [3] and it has been reported that this is present in up to 55–80% of extremely preterm infants [4, 5]. It can either be seen in isolation or together with other white matter changes, such as reduced volume, cystic lesions, and delayed myelination, and it has been suggested that DEHSI reflects diffuse white matter injury [3, 5, 6]. Although visual inspection of DEHSI is a subjective measure, diffusion MRI studies have shown that preterm infants with DEHSI display changes in diffusion measures, indicating altered white matter microstructure, when compared to infants without DEHSI [4, 7, 8].

Children born extremely preterm often have neurological, cognitive and motor difficulties [9–11] and it is important to identify whether DEHSI is related to later neurodevelopmental problems. Several studies have evaluated the influence of DEHSI on development in preterm infants at toddler age and contradictory results have been reported. While some studies showed an association between DEHSI and developmental outcomes [5, 12–14], others did not find any relationship to outcome [8, 15–18]. To our knowledge, there are no previous studies that have explored the relationship between the occurrence of DEHSI in the absence of other white matter abnormalities, and long-term outcomes in extremely preterm born children. Our aim was to investigate whether DEHSI was related to neurodevelopmental outcome at 6.5 years of age in children born extremely preterm.

## Materials and Methods

### Study population

Extremely preterm children born before 27 weeks of gestation in Stockholm, Sweden, between January 1st 2004 and March 31st 2007 were included in this study and 108 underwent MRI scans at term-equivalent age. Their developmental outcome was assessed at 6.5 years of age and the examiners were blinded to the MRI results.

In order to study the associations between DEHSI and outcome, 17 children were excluded because there was evidence of parenchymal brain lesions on neonatal cranial ultrasound: intraventricular hemorrhage grade III-IV (n = 11), cystic periventricular leukomalacia (n = 2), periventricular hemorrhagic infarction (n = 1), cysts (n = 2) and hydrocephalus (n = 1). We also excluded MRI scans that showed nodular heterotopia (n = 2) and those with moderate or severe white matter abnormalities (n = 4) around term equivalent age. Two infants were excluded because their gestational age at scan was over 44 weeks. The final sample consisted of 83 children.

The regional ethics committee in Stockholm gave approval for the study and written parental consent was obtained for all participants.

### Perinatal information and cranial ultrasound

The children´s parents reported their educational level. Above high school was considered as high level of education.

The following perinatal information was collected; clinical Risk Index for Babies (CRIB) defined by using birth weight, gestational week, congenital malformations, minimum base excess in first 12 hours and minimum and maximum appropriate fraction of inspired oxygen in the first 12 hours [19]. A neonatologist trained in ultrasound performed the cranial ultrasounds, and the first was done within the first three days after birth, then at least once a week until 27^th^ week, every second week until discharge and at term equivalent age. All children with signs of pathology on ultrasound, clinically ill or unstable, were scanned more frequently, depending on the severity of the pathology. Intraventricular hemorrhage was graded according to Papile [20].

### MRI data acquisition

The MRI scans were performed on a Philips Intera 1.5T MRI system (Philips International, Amsterdam, The Netherlands) at term equivalent age, with a median gestational age of 40.42 weeks (range 39.14–43.28). The MRI protocol consisted of a sagittal T1-weighted turbo spin echo sequence, an axial inversion recovery sequence and an axial T2-weighted sequence. Details of the MRI protocol have previously been described [4].

### Visual assessment of MRI

The presence of DEHSI and other white matter abnormalities was assessed and scored by visual inspection of the T1-weighted and T2-weighted images. DEHSI was defined as high signal intensity in the periventricular and subcortical white matter on T2-weighted images [3]. The presence of DEHSI was evaluated in the frontal and occipital regions bilaterally. Two experts (a pediatric neuroradiologist, MM and a pediatrician, BS) evaluated the T2 weighted images once each on separate occasions independently of one another. The inter-observer variability was measured in all the 66 scans using the Kappa statistics where a kappa of 1 indicates perfect agreement, whereas a kappa of 0 indicates agreement equivalent to chance. The concordance between assessors was 91%, kappa = 0.807; p<0.005 which is interpreted as a good agreement [21].

Other white matter abnormalities were described according to a previously published protocol [22, 23] that qualitatively evaluated thinning of the corpus callosum, stage of myelination, reduction in white matter volume, white matter signal abnormalities, ventricular dilation, and cysts. Based on this assessment, the dataset was split into four groups, namely no, mild, moderate or severe white matter abnormalities. Only the normal and mild white matter abnormality groups were included in the study. According to the same scoring system, gray matter was evaluated and divided into two groups; normal and abnormal gray matter [22, 23]. Cerebellar injuries were evaluated according to a standard clinical radiologic assessment.

### Neurodevelopmental outcome

#### Neurological assessment

Two pediatricians (JB and UA) with training in neurological examination took turn to examine the children´s neurology profile. The neurological assessment was performed using a modified Touwen examination [24], which enables the assessment of minor neurological dysfunction (MND). This provides a description of the child’s neurological profile, including difficulties in muscle tone regulation, balance and coordination, reflexes and nerve function of the face and eyes. Minor neurological dysfunction can be categorized into normal, MND 1 (no clinical significance) and MND 2 (clinically significant dysfunction that has been associated with cognitive and behavioral problems) [24, 25].

Cerebral Palsy (CP), was diagnosed based on the Surveillance of Cerebral Palsy in Europe working group criteria [26].

#### Motor function and cognitive assessment

Motor function was assessed with the Movement Assessment Battery for Children 2 (M-ABC-2) [27], which assesses manual dexterity, aiming and catching, and balance skills. Each subtest has a standard score, and scores are summarized into a total composite score, total standard score, and percentile. Borderline motor problems are indicated if the score falls between the 5^th^ and 15^th^ percentile, and definite motor problems are associated with scores ≤ 5^th^ percentile. We used composite scores for analyses. General cognitive abilities were assessed with the Wechsler Intelligence Scale for Children—Fourth Edition (WISC-IV) [28]. The subtests for speed, perceptual functioning, verbal functioning, and working memory respectively are summarized to a total raw score. Raw scores are transformed into standard scores and then to intelligence quotient scores. The mean standard score in a normal population is an intelligence quotient of 100, with a standard deviation of 15.

#### Visual-motor integration (VMI) evaluation

Visual-motor integration was assessed with the Beery VMI test 6^th^ edition [29]. This paper and pencil task consists of 30 figures that the child has to copy. The raw score is transformed into an age-adjusted standard score with a mean of 100 and a standard deviation of 15 and 70 is considered the cut-off for clinically relevant problems.

#### Behavioral evaluation

The parents’ version of the Strengths and Difficulties Questionnaire (SDQ) [30, 31] was used to screen for behavioral difficulties. Items included in the SDQ are: emotional symptoms (normal range 0–3), conduct problems (0–2), hyperactivity/attention (0–5) and peer relationship problems (0–2). These items are then summed into a total raw score ranging from 0–40 and a score of 0–13 is considered normal. Prosocial behavior is scored separately and 6–10 is regarded as a normal score.

### Statistical analysis

Statistical analyses were performed with PASW Statistics 22.0 (SPSS Inc, Chicago, Illinois, USA). Comparisons between the groups were performed using the Student t-test, Mann-Whitney U-test, ANOVA or Kruskal-Wallis test for continuous variables. Fisher´s Exact test or the Chi-square test was used for dichotomous variables. Analyses were adjusted for birth weight. A general linear model was performed with the scores from the WISC-IV (total score), M-ABC-2 (total score), VMI and SDQ as dependent variables and group status—DEHSI or no DEHSI—as the independent factor. Logistic regression analyses were applied for neurological status—normal, MND 1, MND 2 and CP—as dependent variables. A p-value of <0.05 was the cut-off for statistical significance.

## Results

**Fig 1:**
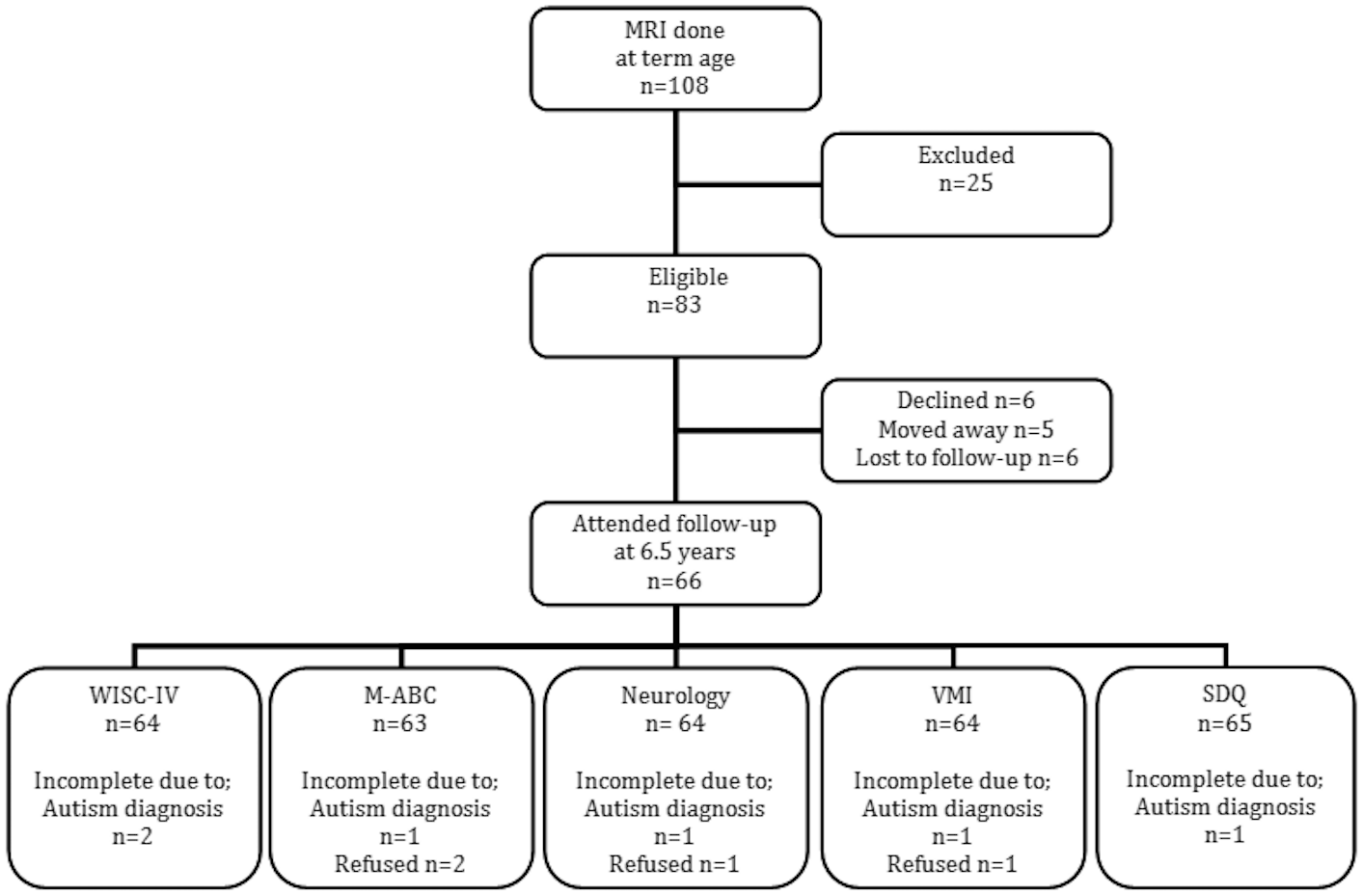
Flow chart showing the study group. Eighty percent of the preterms that performed MRI attended follow-up.

The 6.5-year follow up was performed in 66 (80%) of the 83 the children with neonatal MRI scans and the reasons for the drop-outs are presented in Fig 1. There were no statistically significant differences in perinatal characteristics between the children who did and did not take part in the follow up (Table 1).

**Table 1:** Perinatal characteristics and MRI findings in children attending and not attending follow-up.

	Attended (n = 66)	Did not attend (n = 17)^a^	p-value
**Birth weight (g), mean ± SD**	**822 ± 156**	**756 ± 166**	**0.13**
**Gestational age at birth, weeks, median (range)**	**25.5 (23.1–26.6)**	**25.6 (23.3–26.8)**	**0.95**
**Male gender, n (%)**	**33 (50)**	**12 (71)**	**0.18**
**Small for gestational age, n (%)**	**4 (6)**	**3 (18)**	**0.15**
**Multiple births, n (%)**	**11 (17)**	**4 (24)**	**0.50**
**Prenatal steroids, n (%)**	**60 (91)**	**17 (100)**	**0.34**
**Premature rupture of membranes, n (%)**	**17 (26)**	**6 (35)**	**0.54**
**CRIB score, median (range)**	**5 (1–13)**	**7 (2–11)**	**0.12**
**Postnatal steroids, n (%)**	**11 (17)**	**2 (12)**	**1.00**
**Sepsis, n (%)**	**51 (77)**	**13 (76)**	**1.00**
**Necrotizing enterocolitis Bell´s grade 23, n (%)**	**8 (12)**	**2 (12)**	**1.00**
**Intraventricular hemorrhage I-II, n (%)**	**25 (38)**	**7 (41)**	**0.79**
**Mechanical ventilation (days), median (range)**	**8 (0–43)**	**9 (0–47)**	**0.67**
**Days on CPAP, median (range)**	**39 (13–108)**	**37 (20–58)**	**0.75**
**BPD, oxygen at age 36 weeks, n (%)**	**26 (39)**	**6 (35)**	**1.00**
**Patent ductus arteriosus, Ibuprofen treated, n (%)**	**45 (68)**	**12 (71)**	**1.00**
**Patent ductus arteriosus, surgical ligation, n (%)**	**22 (33)**	**5 (29)**	**1.00**
**Retinopathy of prematurity, laser-treated, n (%)**	**9 (14)**	**6 (35)**	**0.07**
**Gestational age at scan, median (range)**	**40.42 (39.14–43.28)**	**40.70 (38.00–43.42)**	**0.79**
**Normal white matter, n (%)**	**36 (55)**	**14 (82)**	**0.051**
**Mild white matter abnormality, n (%)**	**30 (45)**	**3 (18)**	**0.051**
**Gray matter abnormality, n (%)**	**2 (3)**	**0**	**1.00**
**Cerebellar injury, n (%)**	**3 (5)**	**2 (12)**	**0.27**

Significance value, p<0.05, SD = Standard Deviation, CPAP = Continues Positive Airway Pressure, CRIB score = Clinical Risk Index for Babies, BPD = Bronchopulmonary dysplasia. Sepsis was defined as positive blood cultures or clinical signs of sepsis in association with elevated C-reactive protein or leukocyte count. IVH = intraventricular hemorrhage.

^a^ Of the 17 children not attending 5/17 had DEHSI.

### DEHSI on MRI

**Fig 2:**
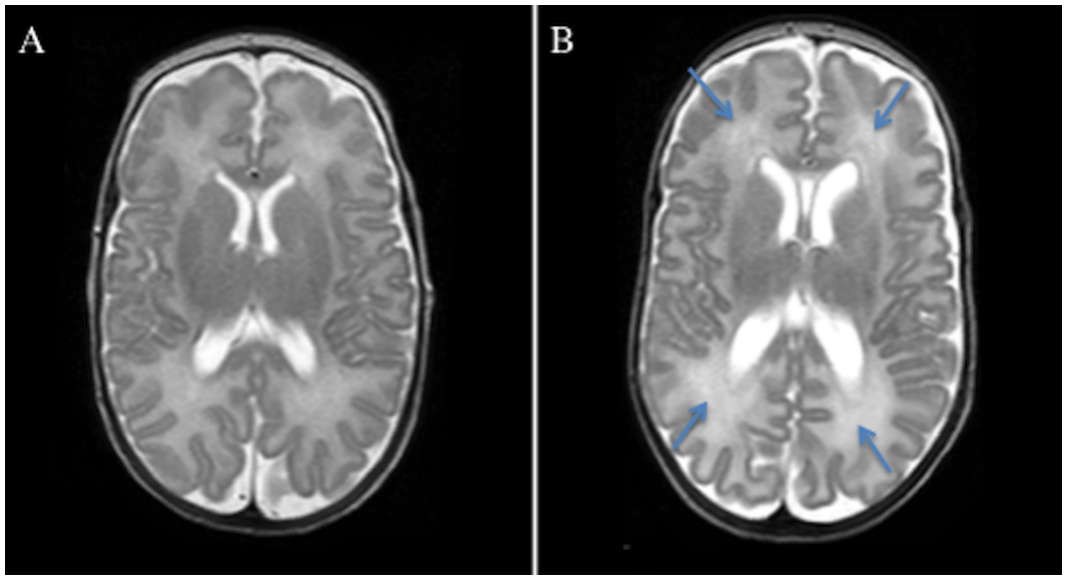
Axial T2-weighted MR images. Normal white and gray matter (A) and diffuse excessive high signal intensity (DEHSI) in the frontal and occipital white matter bilaterally (B). Blue arrows indicate presence of DEHSI.

DEHSI was present (Fig 2) in 39 (59%) of the 66 children assessed at 6.5 years. 37/39 (95%) had bilateral DEHSI and 23/39 (59%) had both frontal and occipital DEHSI (either uni or bilateral). 14 (4%) children had only frontal and two (5%) had only occipital DEHSI. There were no significant differences in terms of neonatal characteristics or neonatal morbidities between the infants with and without DEHSI (Table 2), but the infants with DEHSI tended to have a higher birth weight (p = 0.056).

**Table 2:** Perinatal characteristics and MRI findings in children attending follow-up at 6.5 years.

	DEHSI (n = 39)	No DEHSI (n = 27)	p-value
**Birth weight (g), mean ± SD**	**852 ± 159**	**778 ± 143**	**0.056**
**Gestational age at birth, weeks, median (range)**	**25.5 (23.1–26.6)**	**25.6 (23.4–26.6)**	**0.82**
**Male gender, n (%)**	**20 (51)**	**13 (48)**	**1.0**
**Small for gestational age, n (%)**	**1 (3)**	**3 (11)**	**0.30**
**Multiple births, n (%)**	**8 (21)**	**3 (11)**	**0.50**
**Prenatal steroids, n (%)**	**35 (90)**	**25 (93)**	**1.0**
**Premature rupture of membranes, n (%)**	**9 (23)**	**8 (30)**	**0.58**
**CRIB score, median (range)**	**5 (1–13)**	**5 (1–12)**	**0.64**
**Postnatal steroids, n (%)**	**5 (13)**	**6 (22)**	**0.34**
**Sepsis, n (%)**	**30 (77)**	**21 (78)**	**1.0**
**Necrotizing enterocolitis Bell´s grade 2–3, n (%)**	**5 (13)**	**3 (11)**	**1.0**
**Intraventricular hemorrhage I-II, n (%)**	**15 (38)**	**10 (37)**	**1.0**
**Mechanical ventilation (days), median (range)**	**5 (0–43)**	**9 (0–39)**	**0.26**
**Days on CPAP, median (range)**	**41 (13–64)**	**38 (21–108)**	**0.41**
**BPD, oxygen at age 36 weeks, n (%)**	**13 (33)**	**13 (48)**	**0.31**
**Patent ductus arteriosus, Ibuprofen treated, n (%)**	**27 (69)**	**18 (67)**	**1.0**
**Patent ductus arteriosus, surgical ligation, n (%)**	**14 (36)**	**8 (30)**	**0.79**
**Retinopathy of prematurity, laser-treated, n (%)**	**6 (15)**	**3 (11)**	**0.73**
**Gestational age at scan, median (range)**	**40.43 (39.29–42.86)**	**40.42 (39.14–43.28)**	**0.51**
**Normal white matter, n (%)**	**11 (28)**	**25 (93)**	**<0.001**
**Mild white matter abnormality, n (%)**	**28 (72)**	**2 (7)**	**<0.001**
**Gray matter abnormality, n (%)**	**2 (5)**	**0**	**0.51**
**Cerebellar injury, n (%)**	**3 (8)**	**0**	**0.26**

Significant value, p<0.05, SD = Standard Deviation, CPAP = Continues Positive Airway Pressure, CRIB score = Clinical Risk Index for Babies, BPD = Bronchopulmonary dysplasia. Sepsis was defined as positive blood cultures or clinical signs of sepsis in association with elevated C-reactive protein or leukocyte count. IVH = intraventricular hemorrhage.

### DEHSI and association with outcome

Neurology, motor performance, cognitive, visual-motor or behavioral outcome at 6.5 years did not differ between the children with and without DEHSI (Table 3).

**Table 3:** Outcomes at 6.5 years in children with and without DEHSI.

	DEHSI (n = 39)	No DEHSI (n = 27)	p-value
**Age at assessment (months), uncorrected, median (range)**	**77.22 (75.29–82.18)**	**77.20 (76.03–86.33)**	**0.40**
**Neurology**	**n = 37**	**n = 27**	
**Normal, n (%)**	**19 (51)**	**11 (41)**	
**MND 1, n (%)**	**15 (41)**	**12 (44)**	
**MND 2, n (%)**	**2 (5)**	**1 (4)**	
**CP-diagnosis, n (%)**	**1 (3)**	**3 (11)**	
**Normal/MND 1, n**	**19/15**	**11/12**	**0.60**^a^
**Normal/MND 2+CP, n**	**19/3**	**11/4**	**0.41**^b^
**Motor function**	**n = 37**	**n = 26**	
**Total test score, mean ± SD**	**68 ± 15**	**66 ± 19**	**0.60**
**Manual dexterity, median (range)**	**23 (8–35)**	**26 (7–38)**	**0.92**
**Aiming and catching, mean ± SD**	**17 ± 5**	**18 ± 5**	**0.48**
**Balance, median (range)**	**29 (7–36)**	**28 (12–36)**	**0.28**
**Cognition**	**n = 38**	**n = 26**	
**Total scaled score, mean ± SD**	**87 ± 20**	**82 ± 18**	**0.33**
**Speed, mean ± SD**	**16 ± 5**	**15 ± 4**	**0.53**
**Perceptual, mean ± SD**	**27 ± 7**	**26 ± 7**	**0.59**
**Working memory, mean ± SD**	**14 ± 4**	**13 ± 3**	**0.39**
**Verbal, mean ± SD**	**29 ± 8**	**27 ± 8**	**0.27**
**Visual Motor Integration (VMI)**	**n = 37**	**n = 27**	
**Standard score, mean ± SD**	**92 ± 14**	**88 ± 11**	**0.23**
**Strengths and Difficulties (SDQ)**	**n = 39**	**n = 26**	
**Overall raw score, median (range)**	**7 (0–27)**	**10 (0–26)**	**0.59**
**Emotional problems, median (range)**	**1 (0–9)**	**2 (0–8)**	**0.49**
**Conduct problems, median (range)**	**1 (0–7)**	**1 (0–7)**	**0.41**
**Hyperactivity, median (range)**	**3 (0–10)**	**3 (0–9)**	**0.90**
**Peer problems, median (range)**	**1 (0–6)**	**1 (0–5)**	**0.70**
**Prosocial, median (range)**	**9 (4–10)**	**9 (4–10)**	**0.98**

Significant value, p<0.05, SD = Standard Deviation, MND = Minor Neurological Dysfunction, CP = Cerebral Palsy.

^a^ Comparing normal neurology and MND 1 and

^b^ Comparing normal neurology and MND 2+CP.

After adjustment for birth weight in general linear models the non-significant results remained between the groups in motor function, F(1.60) = 0.02; p = 0.9, cognition, F(1.61) = 0.14; p = 0.7, VMI, F(1.61) = 5.2; p = 0.48 and behavior, F(1.62) = 0.13; p = 0.72. DEHSI was not associated with neurology condition when birth weight was taken into account. The presence of DEHSI was not associated with MND 1 (odds ratio, 0.77; 95% confidence interval, 0.26–2.28, p = 0.64) or MND2/ CP (odds ratio, 0.67; 95% confidence interval, 0.11–4.24, p = 0.67).

There were no statistical differences in outcomes between those with no DEHSI, both frontal and occipital DEHSI and only frontal or occipital DEHSI (neurology, p = 0.57; M-ABC total score, p = 0.72; WISC total score, p = 0.56; VMI standard score, p = 0.56; SDQ overall score, p = 0.86).

The analyses were run with and without preterm infants with cerebellar injuries and gray matter abnormalities and there were still no significant differences between the groups.

Parental educational level did not differ between the groups (p = 0.63). Four children with DEHSI and three children without DEHSI had been diagnosed with autism at the time of the assessment.

## Discussion

DEHSI is a frequent imaging finding [3–5] in children born prematurely. The findings of this study show that isolated DEHSI, regardless of severity, on neonatal MRI scan is not associated with neuromotor, cognitive, visual-motor integration or behavioral outcome at 6.5 years of age.

The origin of these signal abnormalities is unclear and there are conflicting results when it comes to associations with outcome. The subjective nature of the DEHSI finding may play a role in these variable outcomes. DEHSI is defined based on visual assessment of the T2-weighted images and therefore it is accompanied by a high variability associated with this subjective description. In this regard, quantitative measures derived from DTI using objectively defined DEHSI constitute promising methodologies, that may provide less subjective measures that reflect white matter microstructure [12]. In the present cohort we have previously reported altered diffusion in the group with isolated DEHSI compared with preterms without DEHSI [4]. We also had good inter-observer reliability in this study.

Our results are consistent with studies that did not find associations between DEHSI and outcomes in toddlers [8, 15, 17, 18]. Kidokoro et al divided DEHSI into four groups depending on the number of regions that DEHSI was found in. In addition, a small group of 13 children where the margins of posterior periventricular crossroad regions were invisible due to DEHSI, was described. They did not find any correlation between degree of DEHSI and developmental outcomes at age two years. However, lower Mental Developmental Index (MDI in the Bayley Scales of Infant Development-II) was found in infants with invisible posterior crossroads than in those without. Gestational age and exposure to postnatal corticosteroids differed significantly between the groups, but adjustments for this in the analysis were not made, which might have influenced the results [32].

Iwata et al studied children born very preterm and published two studies on DEHSI in relation to outcomes at school age [13, 14]. They found that the prevalence of DEHSI was low on T2-weighted images (17%) and reported a lower full scale IQ in six-year-old children with DEHSI when they adjusted for their corrected age at the time of the scan. On the other hand, DEHSI was not related with cognition at nine years of age. In addition, they used fluid-attenuated inversion recovery (FLAIR) imaging and found that children with abnormal white matter on FLAIR had lower IQ compared to the children with normal white matter on FLAIR at both six and nine years of age. However, they did not look at isolated DEHSI and children who had focal lesions or moderate white matter abnormalities in the cohort could potentially have influenced the results [13, 14].

It has been discussed whether DEHSI is an indication of diffuse white matter injury or delayed maturation of the white matter. Using diffusion MRI, our group and others have previously shown that infants with DEHSI have lower fractional anisotropy (FA) and higher apparent diffusion coefficient (ADC) in the white matter [4, 6, 7, 32, 33] indicating altered white matter organization. Some studies have shown that neonates with DEHSI have similar FA and ADC values as preterm infants with overt white matter injuries, suggesting that DEHSI represents diffuse white matter injuries [6, 33, 34]. However, Hart et al showed that increased ADC values in the DEHSI regions were not related to neurodevelopmental outcomes in toddlers who were born preterm [15].

In contrast, some studies in toddlers have found an association between DEHSI and adverse outcomes. In a study by Dyet at al, the number of children with isolated DEHSI without other white matter abnormalities were low, and although there was a statistically significant difference in the Developmental Quotients on the Griffiths Scales between children with and without DEHSI, these differences were only one standard deviation below the mean and still within the normal range [5].

Parikh et al found no correlation between visually assessed DEHSI and cognition and language using the Bayley Scales of Infant Development-II at the age of two years. However, when they quantified DEHSI volumes with diffusion tensor imaging they found a correlation with the two-year outcome in a small number of infants, suggesting that DEHSI is a pathologic sign [12].

Krishnan et al found an association between higher ADC values in the white matter and poorer developmental outcome at two years of age in preterm born children. They argued that children with DEHSI often show elevated ADC values in the white matter, and that their findings therefore supported a relation between DEHSI and neurodevelopmental outcome [35].

In summary, a few studies have suggested an association between DEHSI and toddler outcomes, but there is little support for an association to school age outcomes, which may indicate that DEHSI represents delayed maturation.

Kostovic et al published histological findings of 21 postmortem human brains, ranging in age from 26 weeks of gestation to 6.5 years of age, and compared them to 30 MRI images from age-matched cases. They concluded that when DEHSI could be visually assessed on MRI, the image represented the remnants of the subplate at term age, pointing out that the subplate subsequently disappeared and was replaced by myelinated axons [36]. This supports the theory that DEHSI and its associated altered diffusion measures represent delayed maturation rather than white matter injuries.

To our knowledge, our study is the largest published prospective follow-up study in extremely preterm infants with isolated DEHSI. Imaging was performed at an adequate age for visually assessing DEHSI and the follow-up rate at 6.5 years was high. Even if our sample size was moderate, we believe that our well-defined cohort, and the fact that we focused exclusively on isolated DEHSI findings, makes our results highly clinically relevant.

## Conclusions

This study showed that the presence of qualitatively defined DEHSI on neonatal MRI was not associated with developmental outcomes of children who were born extremely preterm when they reached 6.5 years of age. The results support the theory that DEHSI reflects delayed maturation rather than white matter injuries. Better methods to objectively define DEHSI are necessary before we can make the definitive conclusion that DEHSI is not related to neurodevelopmental outcomes. There is a need for a reliable and objective tool that can predict later impairment in extremely preterm children, but isolated DEHSI by visual inspection does not provide useful information in this context.

## Supporting Information

S1 File DEHSI scoring and examination results. (XLSX)
